# Supplementary material for: Bursts of amino acid replacements in protein evolution
Source: R Soc Open Sci. 2019 Mar 27;6(3):181095. doi: 10.1098/rsos.181095 (PMC6458383; doi:10.1098/rsos.181095)

Supplementary Fig. 1. Reconstructed phylogenetic trees of the analyzed species. Internal edges shorter than 0.005 dS are in bold and edges that harbor bursts of evolution are in red and numbered. Bootstrap values lower than 100 are shown. A: Five Baikal gammarids clades (Naumenko et al, 2007). B: Catarrhini (Rosenbloom *et al.*, 2015).

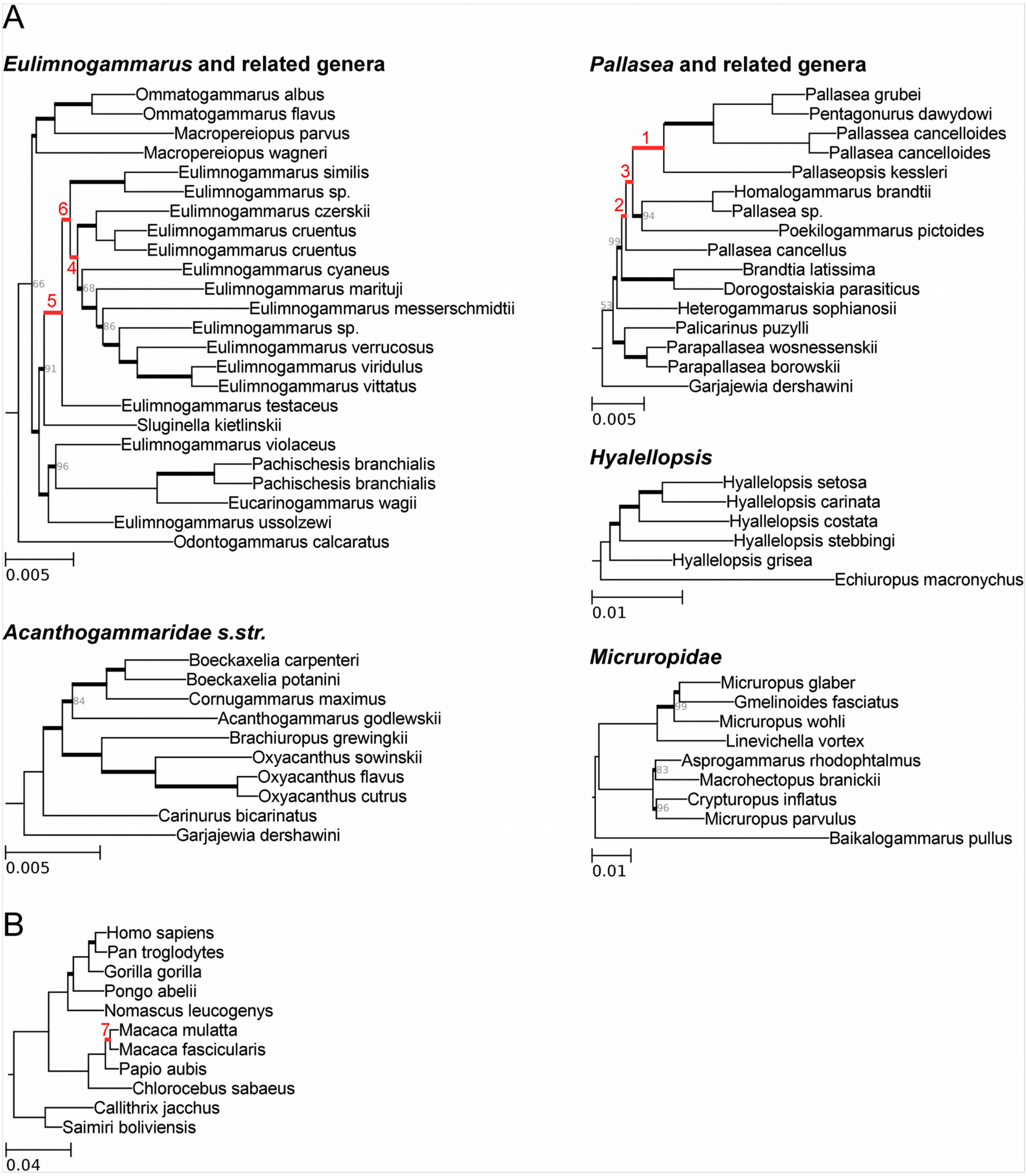

Supplementary Fig. 2: Alignments of gammarid genes containing bursts of substitutions. Post-burst species are in gray. Alleles derived in the adaptive burst are shown in bold.

MRPL22

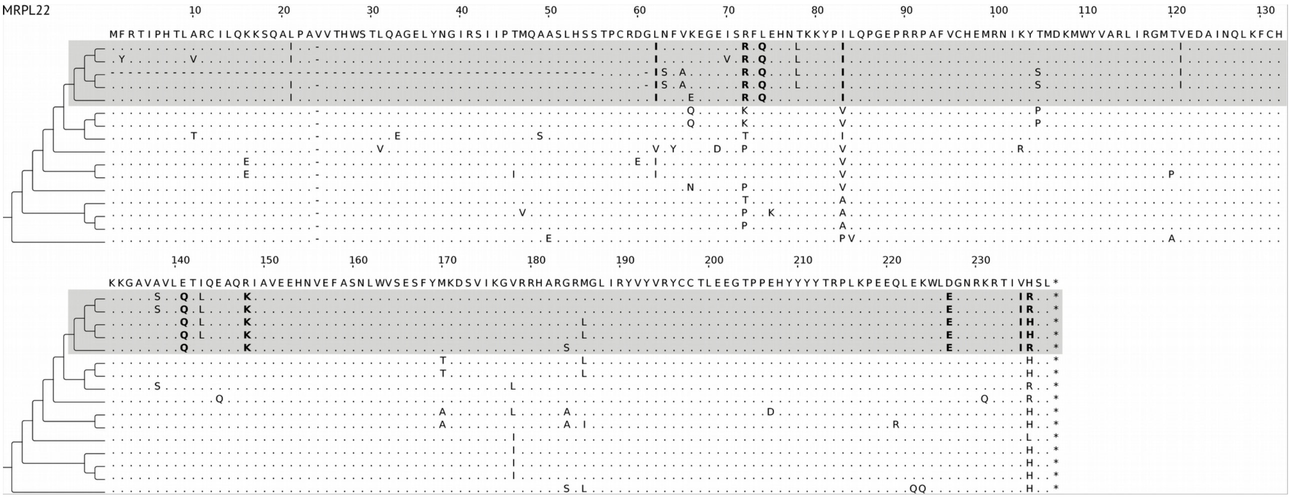

NOP16

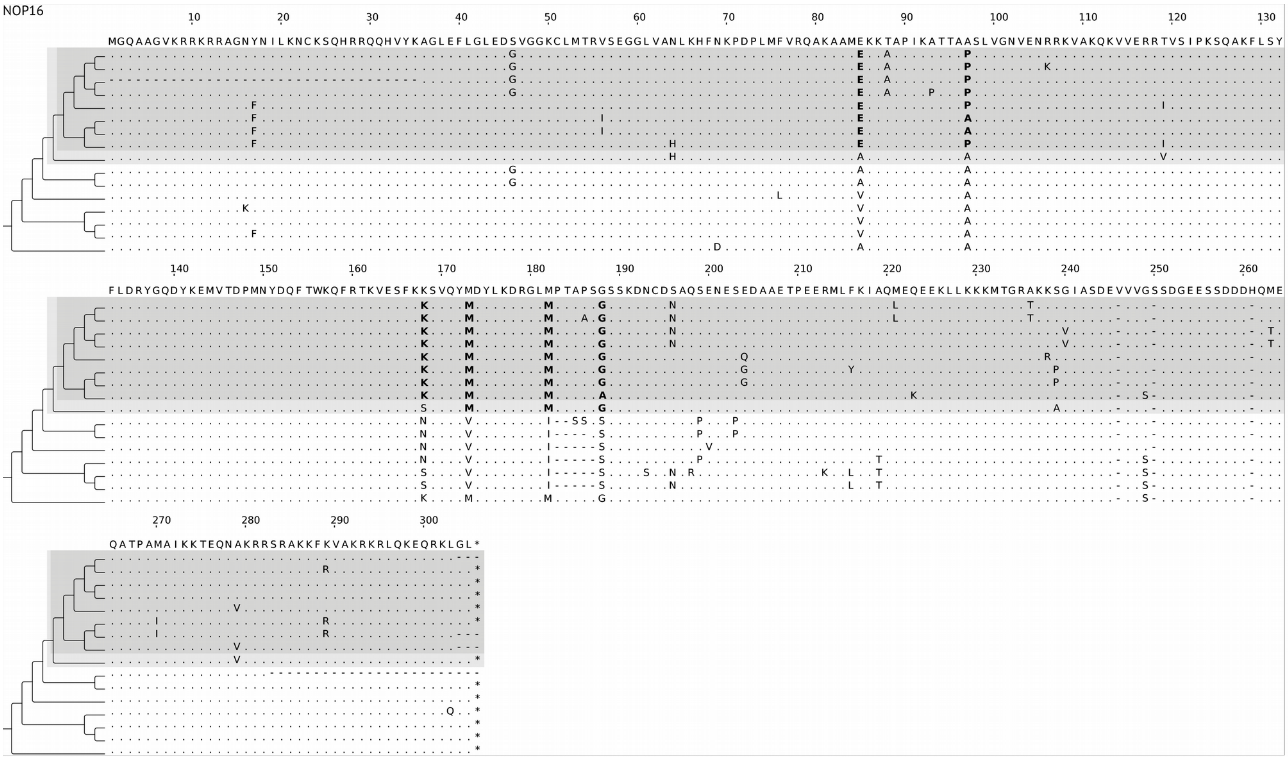

AKR1

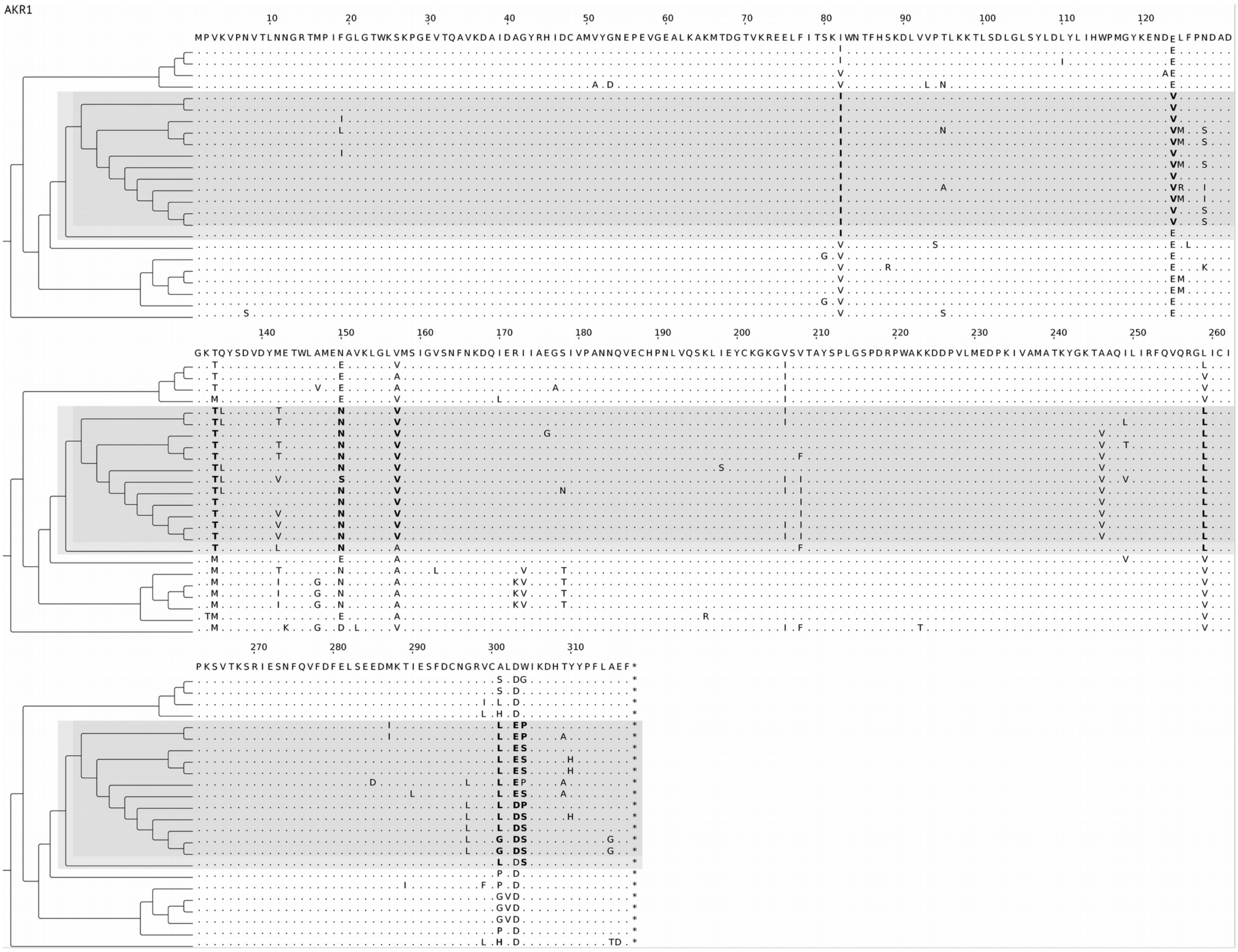

MRPS25

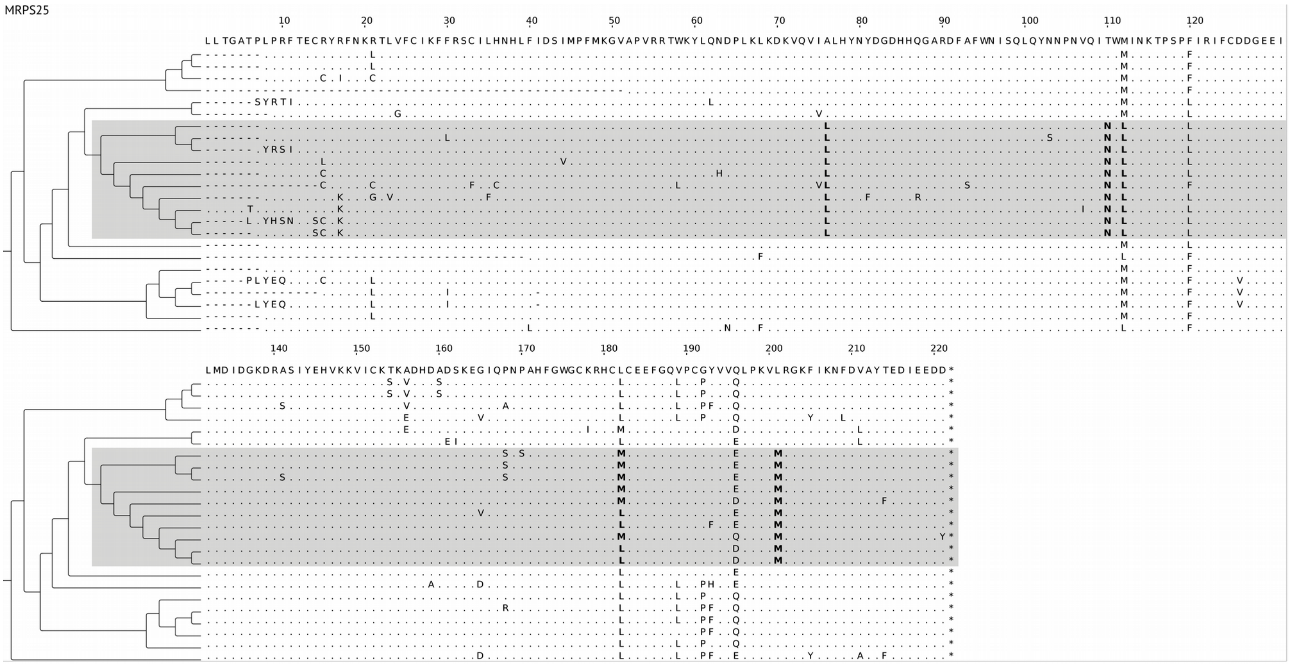

Supplementary Fig. 3. Annotation of gammarids genes in the initial dataset.

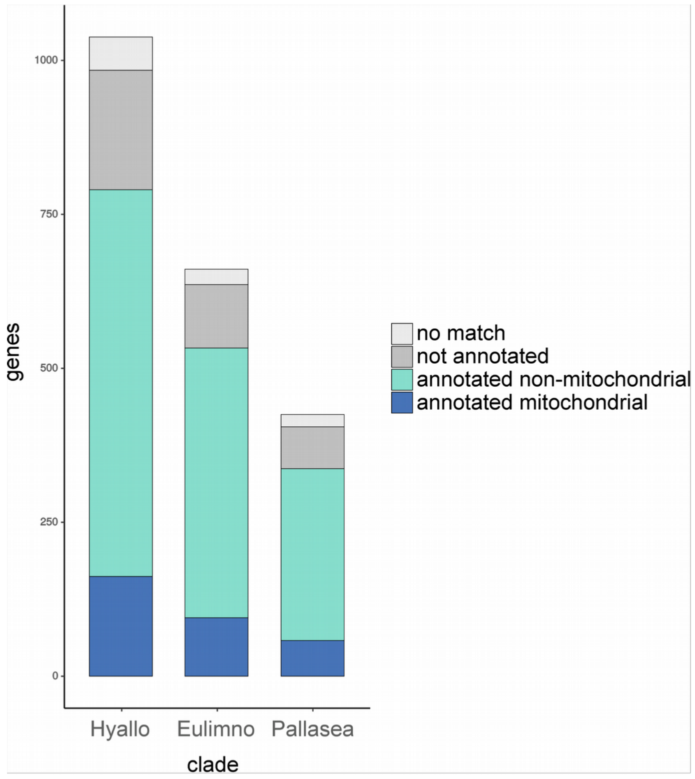

Supplementary Fig. 4. dN values for genes containing adaptive bursts on phylogenetic trees. Numbers and colors correspond to the edge-specific, gene-specific dN divided by the length of the edge in dS. Burst-carrying edges are in bold. A. *Pallasea* and related genera, B. *Eulimnogammarus* and related genera.

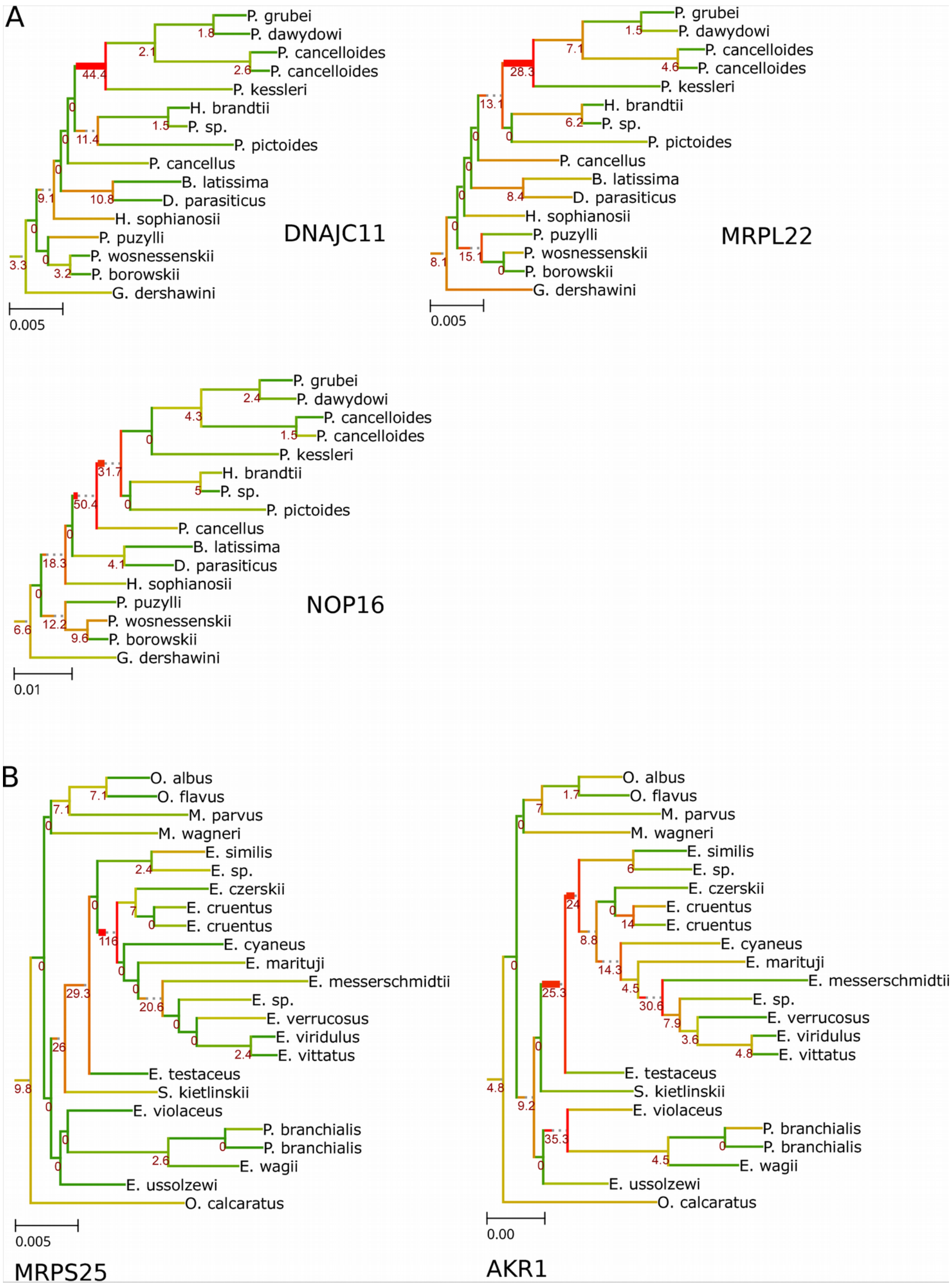

Supplement: Supplementary figures [file rsos181095supp1.pdf]
